# Proof firm downsizing and diagnosis-specific disability pensioning in Norway

**DOI:** 10.1186/1471-2458-13-27

**Published:** 2013-01-11

**Authors:** Bjørgulf Claussen, Øyvind Næss, Leif Jostein Reime, Alastair H Leyland

**Affiliations:** 1 Institute of Health and Society, University of Oslo, P.O.Box 1130, Blindern, N0318, Norway; 2 National Institute of Public Health, Oslo, Norway; 3 MRC/CSO Social and Public Health Sciences Unit, University of Glasgow, Glasgow, UK

**Keywords:** Disability pension, Labour market, Firm downsizing

## Abstract

**Background:**

We wanted to investigate if firm downsizing is related to an increased rate of disability pensions among the former employed, especially for those with musculoskeletal and psychiatric diagnoses, and for those having to leave the firm.

**Methods:**

Statistics Norway provided a linked file with demographic information and all social security grants from the National Insurance Administration for 1992–2004 for all inhabitants in Norway. Our sample was aged 30–55 years in 1995, being alive, employed and not having a disability pension at the end of 2000. Downsizing was defined as percent change in number of employed per firm from 1995 to end 2000. Employment data were missing for 25.6% of the sample.

**Results:**

Disability pension rates in the next four years were 25% higher for those experiencing a 30-59% downsizing than for those not experiencing a reduction of the workforce. 1-29% and 60-100% downsizing did not have this effect. Stayers following down-sizing had higher disability pension rates than leavers. What we have called complex musculoskeletal and psychiatric diagnoses were relatively most common.

**Conclusion:**

Moderate downsizing is followed by a significant increase in disability pension rates in the following four years, often with complex musculoskeletal and psychiatric diagnoses.

## Background

Disability pension (DP) is relatively generous in Norway, giving a pension from 50% to a maximum of 66% of wages before disability, and about 60% for ordinary wages. About 11% of the population aged 18 to 66 years old have a DP [1]. In recent years, this tax-paid social security system comprising all persons living in Norway has been vividly discussed. Being unable to work because of disease, injury or handicap determines eligibility to receive a DP for stably working people, and demands a medical certificate with such a diagnosis. However, 40% of the disabling diagnoses are musculoskeletal, often considered medically unspecific diagnoses like muscle pain or low back pain, and another 30% are psychiatric diagnoses, mostly depression or anxiety with various levels of morbidity [1].

Structural causes of disability pensioning have not been widely studied. Vahtera et al. took advantage of a “natural experiment” in Finnish municipalities during a recession in 1993, and obtained data on more than 22,000 employees before and after downsizing. Those keeping their municipal jobs had increased sickness absence after major downsizing (>18%) compared to minor downsizing (8-18%), had higher mortality rates in the next five years [2], and used more psychotropic drugs [3]. Disability pensioning in the same period among those aged 21–54 years was 7.7 per thousand for those not experiencing downsizing, 13.1 after minor downsizing and 14.9 per thousand after major downsizing, the last figure being 1.81 times as high as for no downsizing, adjusted for a lot of factors [4].

In a register study of all employed persons in Norway, Rege et al. found that firm downsizing 60% or more in 1995–2000 increased the chance of having a DP in 2001 by 24% compared to employees in non-downsizing firms, with a 9% increase after 30-60% downsizing [5]. These results were robust for differences in region, municipality, industry types and other pre-downsizing properties, sick leave rates before 1995, and diagnostic groups according to the medical certificates of disability.

The aim of the present study was to determine diagnosis-specific disability rates in 2001–2004 for the employees of Norwegian firms downsizing in the period 1995–2000, compared to the employees who did not experience downsizing, adjusted for individual factors. Second, we would analyse DP rates among leavers and stayers, expecting that leavers would have a higher disability rate than stayers, and both groups having higher rates than employees in non-downsized firms. We also hypothesised that high disability rates were associated with high rates of non-specific musculoskeletal and psychiatric diagnoses.

## Methods

The used data file "FD-trygd" is a linkage of Statistics Norway's files of demographics with files from the National Insurance Agency (NAV) about disability pensions from 1.1.1992 to 31.12.2004 for all persons living in Norway in the period, a newer edition of the same file used by Rege et al. [5].

We distinguished between leavers and stayers in downsized firms (which are most similar to “workplaces”) in the period 1995–2000: those who left their 1995 firm in the period (leavers) and those who stayed in the same firm until 2000 (stayers). Individual characteristics were recorded in 1995. The chosen sample was aged 30–55 years in 1995, because they were eligible for DP in the whole period, the oldest ones reaching retirement age after 2004 and the youngest ones being old enough to get DP for reasons other than inborn conditions. Those having DP at 1 January 2000 were excluded, and so were those not living in Norway at 31 December 2004. Like Rege et al., we also excluded those working in firms with less than ten employees because firm downsizing will be poorly defined in such small firms.

### Dependent variable: disability pension

Cumulative incidence of disability pensioning from 1 January 2001 to 31 December 2004 was the dependent variable, taken from the NAV file without missing cases. Thus, 5–10 years will pass from having a job in 1995 to DP receipt. All DPs are granted by the NAV in Norway, private insurance and job pension being supplements. Disability pensioning in Norway is always a long-lasting procedure, starting with one year sick-listing and usually followed by 1–3 years of rehabilitation efforts. Thus, those receiving DP in 1995–2000 were excluded because their DP is usually not associated with firm downsizing in the same period. The social security system was little changed in the period 1993–2004 [6].

### Diagnoses

Primary diagnosis on the doctor's certificate states what is the main medical reason for the applicant’s disability. They were coded according to ICD-9, from end 1998 ICD-10, and grouped into “mental”, “musculoskeletal” and “others”. The first two were further divided into “well defined” and “complex conditions”, the last term being newly introduced in psychatric [7] and somatic [8] classifications:

▪Well defined psychiatric diagnoses comprised psychoses, oligophrenia and similar conditions (ICD-9 290–295; 297–299; 317–319; ICD-10 F00-F09; F20-F29, F70-F79).

▪Complex psychiatric diagnoses were depression, anxiety states, personality disorders, and all alcohol and drug diagnoses (ICD-9 296; 300–302; 303–305; 312–316; ICD-10 F10-F19; F30-F69; F80-F99).

▪Well defined musculoskeletal diagnoses were arthritis, osteoathritis and similar (ICD-9 714; 715; 710–713: 716–719; 725–728; 730–739; ICD-10 M05-M06; M15-M19; M00-M04; M20-M39; M55-M78; M80-M99).

▪Complex musculoskeletal diagnoses were myalgia, fibromyalgia, tenosynovitis, low back pain and similar (ICD-9 729; ICD-10 M79; M40-M55).

▪Other somatic diagnoses were all other somatic diagnoses, mostly well-defined according to medical science.

▪A few applications lacked a registered diagnosis from the primary physician or had a symptom diagnoses. They were referred to a specialist whose diagnoses were not recorded.

### Individual independent variables

*Age* in 1995 was adjusted for as a continuous variable.

*Education* at 1995 is important as a predictor for DP [9], and was adjusted for in three categorical values: basic education (9 years or less for the elderly), middle education (10–12 years) and high education (high school or university exams, 13 years +).

*Income* was taxable income given to the Tax Authorities for 1995, grouped in five groups for bivariate analyses and quintilised for regressions.

*Tenure years* were given from 1992 to 1995 in our data file, i.e. a maximum of four years or more, with groups of less than a half year, a half to one year, 1–2 years, 2–4 years and 4 years +, both for bivariate analyses and regressions. This variable was too skewed to be quintilised.

Part time vs. full time work was also tested but had no influence on DP rates, and was not used.

### Firm independent variables

*Firm downsizing* in the period 1995 to 2000 was hypothesized to be an important predictor of disability pensioning in 2001–04 because elderly employees and others with reduced work ability will tend to apply for a DP if they do not get a new job quite soon. We had data on employers (firms) from 1995 to 2000 for 74.4% of the employed persons, and calculated the number of employees in all Norwegian firms in 1995 from the individual variables in FD-trygd. The variable was grouped in five different groups; increasing workforce to no downsizing, 1-29%, 30-59% and 60-100% downsizing.

*Plant size* may be of importance [5]. This variable was grouped for bivariate analyses and quintilised for regressions.

*The proportion of employees with basic education* in the firm was calculated and quintilised.

*Mean income* among the employees in the firm was calculated and quintilised.

*The proportion of women* in the firm was calculated and quintilised.

The percentage of part time employees, official or private firms and the proportion of elderly employees had no influence on DP rates, and were not used in the regressions.

### Missing values

All people entering and leaving a paid job are to be reported to the NAV because of the eligibility rights for social security benefits following paid jobs (but not the 7% of the workforce who are self-employed). Unfortunately, not all firms comply with this rule, leaving 25.6% of the FD-trygd sample not registered with a job in 1995. Of these, only 0.3% were registered without taxable income, 0.2% women and 0.4% men, indicating few females and males without a paid job. Those missing had the same distribution of gender as the whole sample (25.9% females missing and 25.2% males), a higher proportion had low income (52.9% in the two lowest income groups among missing cases and 41.9%. in the sample) and less education (23.1% basic education among missing cases and 15.7%. in the sample). Thus, those missing data about their firm more often belonged to lower social positions. As smaller firms often employ people in such position, this is in accordance with an analysis of Statistics Norway showing that small firms often do not register their employed persons [10].

### Statistics

All analyses were stratified by gender because differences in disability pensioning across gender are substantial [1]. Bivariate associations were tested by chi-square statistics (Tables 1 and 2). Age-adjustments were done in logistic regressions with the whole gender specific sample as the standard population. The odds of getting a DP in 2001–04 were analysed using multilevel logistic regressions adjusting separately for individual (Model 1) and firm characteristics (Model 2), and for both levels combined (Model 3). This was done because the firm variables were aggregated from the individual data, and were not independent for individuals in the same firm (Tables 3, 4, 5 ,6). We used a two level model with random intercepts. In the present paper we report fixed effects for both individual and firm variables, and also the estimated variance between firms (on a log-odds scale, indicating the extent to which firms differ in rates of DP after adjustment for variables included in each model), the intraclass correlation coefficients (which detail the proportion of the variance not explained by variables included in each model that can be attributed to differences between firms), and the total explained variance (R^2^). Model 3 was repeated for five diagnostic groups (Tables 4, 5, 6). All analyses were done in MLwiN version 2.22.

**Table 1:** Frequency of independent variables (vertical per cent) and incidence (horizontal per cent) of a new disability pension in 2001–2004 of inhabitants in Norway aged 30–55 years and being employed on 1 January 1995

**Individual predictors 1995**	**Females (n=665,632)**		**Males (n=701,971)**	
	**Frequency**	**Incidence of DP**	**Frequency**	**Incidence of DP**
Age				
30-39	43.4	2.5	42.3	1.7
40-49	39.2	6.2	39.6	4.5
50-55	17.4	12.4	18.1	10.7
Education				
Basic education	15.7	9.7	15.6	8.2
Middle education	55.1	5.9	56.6	4.6
High education	29.2	3.1	27.8	2.0
Tenure years				
1-182 days	4.6	5.4	4.3	5.4
182-365 days	5.7	5.3	7.0	5.1
1-2 years	8.9	5.2	9.1	5.1
2-4 years	11.6	5.2	10.5	4.7
4 years	58.5	6.0	64.9	4.0
Missing	10.7	5.4	4.1	7.8
Income per year (NOK				
1-20,000	35.7	5.7	11.7	6.4
20,001-100,000	24.7	5.4	9.7	4.3
100,001-200,000	16.5	5.9	13.8	4.4
200,001-400,000	15.4	5.9	31.6	4.3
400,001 +	7.6	5.5	33.0	4.0
Missing	0.2	1.7	0.2	1.8
Firm downsizing				
No	20.3	5.9	17.5	3.9
1-29%	48.6	5.8	50.7	4.1
30-59%	1.3	7.0	1.6	4.8
60-100%	3.9	4.9	4.9	3.8
Missing	25.9	5.3	25.2	5.5
Fate in 1997				
No downsizing	20.3	5.9	17.5	3.9
Leavers at downsizing	31.5	5.2	36.8	3.7
Stayers at downsizing	22.4	6.6	21.6	4.8
Missing	25.8	7.3	24.1	7.7
All	100.0	5.7	100.0	4.4

All incidence differences are statistically significant with p<0.000.

**Table 2:** Age-adjusted odds ratios (95% confidence interval) of getting a new disability pension in 2001–04 of inhabitants in Norway aged 30–55 years and being employed on 1 January 1995 across quintiles of firm characteristics in 1995

	**Females (n=493,029)**	**Males (n=524,958)**
Plant size (employees)	1.03 (1.01-1.02)	1.06 (1.05-1.07)
Mean income	0.96 (0.95-0.96)	0.92 (0.91-0.93)
Part of female employees	1.01 (1.00-1.02)	1.01 (1.00-1.02)
Part with high education	0.92 (0.91-0.93)	0.80 (0.79-0.81)
Part with middle education	1.04 (1.03-1.05)	1.02 (1.01-1.03)
Part with low education	1.07 (1.06-1.08)	1.18 (1.17-1.19)

**Table 3:** Age-adjusted odds ratios for being granted disability pension in 2001–2004 (95% confidence interval) across individual and firm predictors and both individual and firm predictors in two-level logistic regressions

	**Women (n=493,029**			**Men (n=524,958)**		
	**Individual factors**	**Firm factors**	**Both**	**Individual factors**	**Firm factors**	**Both**
	**Model 1**	**Model 2**	**Model 3**	**Model 1**	**Model 2**	**Model 3**
*Individual factors*						
Education (low=1)						
Middle education	0.66 (0.64-0.68)		0.67 (0.65-0.69)	0.82 (0.81-0.83)		0.82 (0.81-0.83)
High education	0.38 (0.36-0.39)		0.38 (0.37-0.40)	0.66 (0.63-0.68)		0.66 (0.64-0.68)
Tenure years (<1/2 year =1)						
½-1	0.95 (0.89-1.02)		0.96 (0.89-1.02)	0.29 (0.28-0.30)		0.29 (0.28-0.31
1-2	0.93 (0.87-0.99)		0.93 (0.88-0.99)	0.95 (0.89-1.03)		0.96 (0.89-1.30)
2-4	0.88 (0.83-0.94)		0.89 (0.84-0.94)	0.85 (0.79-0.92)		0.86 (0.80-0.92)
4+	0.75 (0.71-0.79)		0.76 (0.72-0.80)	0.74 (0.69-0.80)		0.75 (0.69-0.80)
Income	0.96 (0.95-0.97)		0.97 (0.96--0.97)	0.62 (0.58-0.65)		0.62 (0.59/0.66)
*Firm factors*						
Firm downsizing (No =1)						
1-29%		0.88 (0.84-0.93)	0.90 (0.86-0.95)		0.99 (0.94-1.06)	0.99 (0.94-1.06)
30-59%		1.37 (1.27-1.49)	1.32 (1.22-1.43)		1.24 (1.13-1.36)	1.20 (1.09-1.31)
60-100%		1.00 (0.94-1.06)	0.96 (0.91-1.02)		1.11 (1.04-1.18)	1.04 (0.98-1.11)
Plant size		0.98 (0.97-0.99)	1.03 (1.01-1.04)		0.99 (0.97-1.01)	0.99 (0.98-1.01)
Low education part		1.10 (1.08-1.12)	1.04 (1.02-1.05)		1.07 (1.06-1.09)	1.02 (1.00-1.04)
Mean firm income		0.88 (0.87-0.90)	0.91 (0.89-0.92)		0.89 (0.87-0.91)	0.93 (0.92-0.95)
Mean part of females		0.95 (0.94-0.97)	0.99 (0.97-1.01)		0.91 (0.89-0.93)	0.97 (0.95/0.99)
Variance between firms (SE)	0.083 (0.006)	0.091 (0.007)	0.072 (0.006)	0.134 (0.009)	0.227 (0.012)	0.122 (0.009)
Intraclass correlation coefficient	0.024	0.027	0.021	0.039	0.064	0.036
Explained variance (R2)	0.155	0.137	0.157	0.251	0.196	0.253

**Table 4:** Age-adjusted odds ratios for disability pension in 2001–2004 (95% confidence interval) across individual and firm predictors in two-level logistic regressions for leavers and stayers in downsized firms compared to employees in non-downsizing firms

	**Women**		**Men**	
	**Leavers**	**Stayers**	**Leavers**	**Stayers**
	**(n=219,350)**	**(n=197,612)**	**(n=225,358)**	**(n=173,610)**
*Individual factors*				
Education (low=1)				
Middle education	0.67 (0.64-0.70)	0.67 (0.64-0.70)	0.69 (0.66-0.73)	0.65 (0.62-0.69)
High education	0.37 (0.35-0.39)	0.38 (0.36-0.41)	0.30 (0.28-0.33)	0.31 (0.29-0.34)
Tenure years (<1/2 year =1)				
½-1	0.93 (0.83-1.03)	0.97 (0.87-1.09)	1.00 (0.90-1.12)	0.98 (0.86-1.12)
1-2	0.95 (0.87-1.05)	0.95 (0.85-1.05)	0.85 (0.76-0.95)	0.94 (0.83-1.07)
2-4	0.89 (0.81-0.97)	0.91 (0.82-1.00)	0.77 (0.69-0.86)	0.80 (0.71-0.91)
4+	0.76 (0.70-0.82)	0.78 (0.72-0.85)	0.65 (0.59-0.71)	0.67 (0.61-0.75)
Income (lowest quintile=1)	0.97 (0.96-0.99)	0.97 (0.96-0.99)	0.82 (0.81-0.84)	0.83 (0.81-0.84)
*Firm factors*				
Firm downsizing (none=1)				
1-29%	0.85 (0.79-0.92)	0.96 (0.90-1.03)	0.85 (0.78-0.93)	1.01 (0.94-1.09)
35-59%	1.22 (1.10-1.35)	1.44 (1.20-1.72)	1.10 (0.98-1.23)	1.53 (1.27-1.83)
60-11%	0.91 (0.85-0.97)	1.31 (1.13-1.51)	0.98 (0.91-1.05)	1.69 (1.47-1.94)
Plant size (lowest quintile=1)	1.03 (1.01-1.05)	1.01 (0.99-1.03)	1.00 (0.98-1.03)	0.98 (0.96-1.01)
Low education part (lowest quintile=1)	1.04 (1.03-1.06)	1.04 (1.02-1.06)	1.06 (1.04-1.08)	1.04 (1.02-1.07)
Mean firm income (lowest quintile=1)	0.91 (0.89-0.93)	0.91 (0.90-0.93)	0.93 (0.91-0.94)	0.91 (0.90-0.93)
Mean part of females (lowest quintile=1)	0.96 (0.94-0.99)	0.98 (0.96-1.01)	0.88 (0.86-0.91)	0.93 (0.90-0.96)
Variance between firms (SE)	0.049 (0.007)	0.053 (0.007)	0.124 (0.014)	0.126 (0.014)
Intraclass correlation coefficient	0.015	0.016	0.036	0.037
Explained variance (R2)	0.207	0.193	0.262	0.240

**Table 5:** Age-adjusted odds ratios for diagosis-specific disability pension in 2001–2004 (95% confidence interval) across both individual and firm predictors in two-level logistic regressions

	**Complex MS**	**Well-def. MS**	**Complex psych.**	**Well-def. psych**	**Other somatic**
	**(n=473,605)**	**(n=471,840)**	**(n=470,471)**	**(n=465,167)**	**(n=476,974)**
*Individual factors*					
Education (low =1)					
Middle education	0.65 (0.62-0.69)	0.60 (0.57-0.62)	0.76 (0.71-0.82)	0.71 (0.58-0.87)	0.75 (0.71-0.78)
High education	0.27 (0.25-0.29)	0.21 (0.19-0.23)	0.63 (0.58-0.68)	0.49 (0.38-0.62)	0.51 (0.48-0.54)
Tenure years (<1/2 year =1)					
½-1	0.96 (0.82-1.11)	0.89 (0.78-1.02)	1.10 (0.95-1.28)	0.90 (0.65-1.23)	0.93 (0.83-1.04)
1-2	0.93 (0.82-1.09)	0.96 (0.80-1.09)	1.01 (0.88-1.16)	0.60 (0.44-0.83)	0.90 (0.81-0.99)
2-4	0.90 (0.79-1.03)	0.94 (0.84-1.06)	0.79 (0.69-0.91)	0.67 (0.50-0.90)	0.91 (0.82-1.00)
4+	0.82 (0.79-1.03)	0.80 (0.72-0.89)	0.71 (0.63-0.79)	0.38 (0.30-0.49)	0.77 (0.71-0.84)
Income	0.94 (0.92-0.96)	0.94 (0.92-0.95)	1.02 (0.99-1.04)	0.99 (0.94-1.06)	0.98 (0.96-0.99)
*Firm factors*					
Firm downsizing (no =1)					
1-29%	0.88 (0.78-0.99)	0.94 (0.84-1.04)	0.99 (0.87-1.13)	0.70 (0.55-1.68)	0.99 (0.91-1.09)
30-59%	1.30 (1.09-1.55)	1.14 (0.97-1.36)	1.37 (1.13-1.66)	1.29 (0.86-2.11)	1.33 (1.16-1.52)
60-100%	0.88 (0.78-1.01)	0.99 (0.88-1.11)	0.99 (0.86-1.13)	1.25 (0.95-1.94)	0.93 (0.84-1.02)
Plant size	0.99 (0.97-1.03)	1.00 (0.97-1.03)	1.04 (0.99-1.06)	1.06 (0.98-1.12)	1.00 (0.98-1.03)
Low education part	1.07 (1.04-1.11)	1.05 (1.02-1.08)	0.99 (0.96-1.03)	1.13 (1.00-1.14)	1.01 (0.99-1.04)
Mean firm income	0.92 (0.88-0.96)	0.91 (0.87-0.94)	0.89 (0.85-0.94)	0.96 (0.84-1.01)	0.95 (0.92-0.98)
Part of females	1.03 (0.99-1.08)	0.99 (0.96-1.04)	0.94 (0.91-0.99)	0.92 (0.83-1.02)	0.99 (0.96-1.02)
Variance between firms (SE)	0.074 (0.013)	0.104 (0.013)	0.109 (0.017)	0.186 (0.080)	0.055 (0.008)
Intraclass correlation coefficient	0.022	0.031	0.032	0.054	0.016
Explained variance (R2)	0.265	0.259	0.113	0.052	0.172

Woman.

**Table 6:** Age-adjusted odds ratios for diagosis-specific disability pension in 2001–2004 (95% confidence interval) across both individual and firm predictors in two-level logistic regressions

	**Complex MS (n=506,453)**	**Well-def. MS (n=507,853)**	**Complex psych (n=506,979)**	**Well-def. psych. (504,141)**	**Other somatic (n=515,864)**
*Employed person factors*					
Education (low=1)					
Middle education	0.58 (0.55-0.62)	0.58 (0.55-0.64)	0.80 (0.73-0.86)	0.70 (0.60-0.82)	0.68 (0.65-0.71)
High education	0.14 (0.12-0.16)	0.12 (0.11-0.15)	0.54 (0.47-0.58)	0.46 (0.37-0.56)	0.34 (0.32-0.36)
Tenure years (<1/2 year =1)					
½-1	0.96 (0.82-1.13)	1.09 (0.91-1.31)	0.95 (0.81-1.12)	0.91 (0.72-1.14)	0.95 (0.85-1.05)
1-2	0.95 (0.81-1.11)	0.99 (0.83-1.19)	0.81 (0.69-0.96)	0.68 (0.54-0.86)	0.85 (0.77-0.94)
2-4	0.75 (0.64-0.88)	0.85 (0.71-1.02)	0.72 (0.61-0.85)	0.51 (0.39-0.66)	0.79 (0.71-0.87)
4+	0.67 (0.59-0.77)	0.60 (0.52-0.70)	0.62 (0.54-0.71)	0.38 (0.31-0.46)	0.67 (0.62-0.73)
Income	0.84 (0.83-0.87)	0.85 (0.83-0.85)	0.77 (0.75-0.79)	0.57 (0.55-0.60)	0.85 (0.84-0.86)
*Firm factors*					
Firm downsizing (no =1)					
1-29%	1.02 (0.90-1.16)	1.18 (1.02-1.37)	0.88 (0.76-1.02)	0.88 (0.67-1.16)	0.98 (0.90-1.06)
30-59%	1.26 (1.03-1.54)	1.19 (0.93-1.52)	1.23 (0.98-1.53)	0.94 (0.60-1.50)	1.21 (1.07-1.37)
60-100%	1.15 (1.00-1.32)	0.99 (0.83-1.18)	1.01 (0.76-1.13)	1.12 (0.86-1.45	1.03 (0.94-1.12)
Plant size	0.98 (0.94-1.02)	0.98 (0.93-1.02)	1.02 (1.02-1.09)	1.02 (0.94-1.11)	1.00 (0.98-1.03)
Low education part	1.06 (1.02-1.10)	1.04 (0.99-1.09)	1.00 (1.06-1.13)	1.04 (0.96-1.13)	1.03 (1.00-1.05)
Mean firm income	0.90 (0.86-0.94)	0.89 (0.85-0.94)	0.95 (0.84-0.91)	0.86 (0.78-0.94)	0.93 (0.91-0.96)
Part of females	0.98 (0.93-1.03)	0.88 (0.83-0.94)	1.06 (0.82-0.91)	1.02 (0.92-1.12)	0.96 (0.93-0.99)
Variance between firms (SE	0.204 (0.027)	0.161 (0.031)	0.296 (0.036)	0.240 (0.080)	0.088 (0.011)
Intraclass correlation coefficient	0.058	0.047	0.083	0.068	0.088
Explained variance (R2)	0.314	0.388	0.146	0.189	0.026

Men.

## Results

Of all 1,367,603 employees aged 30–55 years in 1995 and eligible in the four years 2001–2004, 5.7% of females and 4.4% of males got a DP (Table 1). Receipt of pension increased with age and decreased with increasing education and income. Tenure years had a small impact bivariately. The small group of employees who experienced 30-59% downsizing had higher DP rates than all others. Bivariately, stayers after downsizing had a higher DP rate than leavers.

Of firm characteristics, increasing quintiles of plant size and low educated employees seemed to be associated with increasing age-adjusted DP rates, while increasing quintiles of mean income, the proportion of female employees and the proportion with high education were associated with decreases in the DP rate (Table 2).

Diagnoses on the medical certificates were classified in seven groups which are illustrated with more diagnoses in Table 7. Only 1.6% had symptom diagnoses or were missing.

**Table 7:** Disability pensions 2001–04 by diagnosis groups (per cent)

	**Women (n=37,816)**	**Men (n=31,084)**
*Somatic diseases and injuries*	*32.9*	*45.5*
Ischemic heart disease	0.8	1.0
Asthma, KOLS, func. gastrointest. diseases	3.0	4.0
Other internal medicine	15.2	24.7
Neurological conditions	7.0	8.0
Inborn and neonatal errors	0.6	0.3
Skin diseases	1.9	1.5
Injuries	3.1	5.0
*Musculoskeletal well defined*	*19.5*	*17.9*
Myalgia	8.7	1.8
Dorsalgia	10.6	15.1
Fibromyalgia	0.1	0.0
*Psychiatry well defined*	*3.2*	*6.6*
Psychoses	2.0	4.5
Oligophrenia	0.2	0.4
*Psychiatry complex*	*18.4*	*15.6*
Depression	9.9	6.2
Anxiety, personal disorders	7.4	7.9
Misuse	0.4	0.8
*Symptom diagnoses*	*1.3*	*1.4*
*No diagnosis*	*0.3*	*0.3*

These bivariate results were mutually adjusted in logistic regressions (Table 3, 4, 5, 6). Adjusting for only individual variables showed that education and income kept their importance (Table 3 Model 1). Having tenure of more than six months was associated with decreasing DP rates.

Adjusting only for firm variables showed that firms which were downsized by 30 to 59% of the workforce had more disability pensioners 5–10 years later than other firms (Table 3 Model 2). A high proportion of low educated employees in the firm was associated with high DP rates. Low DP rates were associated with high mean employee income and a high proportion of female employees for both genders.

When adjusting for both individual and firm characteristics in Model 3, individual education, income, the firm variables 30-59% downsizing and proportion of low educated still had high rates of DP, while mean firm income and the proportion of females were associated with low rates. Intraclass correlation coefficients were about 70% higher for men than for women, showing that unknown firm characteristics were more important for male than for female DP. For both genders, these coefficients were reduced when both levels were taken into account in Model 3, although the addition of the firm variables did not explain more of the variance than the individual variables alone in Model 1.

These three models were also analysed for *leavers and stayers* compared to those who did not experience any downsizing (Table 4). The two first models showed the same pattern as for all employees in Table 3, so only Model 3 is reported. Both stayers and leavers had higher DP rates than those employees not hit by downsizing but, surprisingly, stayers had higher rates than leavers for both genders, especially males. Intraclass correlation coefficients were the same for stayers and leavers of the same gender but smaller for females than for males, and much smaller for female stayers and leavers than for all employees in Table 3.

For the five diagnostic groups, two-level regressions with a full model were conducted (Tables 5, 6). DP rates due to *complex musculoskeletal disorders* (myalgia, tenosynovitis, low back pain and some others, n = 13,394) were common among employees of firms downsizing 30-59%. Interestingly, highly educated people quite rarely got DP with these diagnoses. Firms with many low educated employees had higher proportions in this disability group. Intraclass correlation coefficients were lower than for all employees, showing that the unexplained firm variance was relatively unimportant for DPs with these diagnoses. Our model explained a relatively high part of the variance.

*Well-defined musculoskeletal diagnoses* (arthritis, osteoarthritis and similar conditions, n = 13,159) were not or were only weakly associated with firm downsizing in contrast to the complex diagnoses above (Tables 5 and 6). Like the first diagnostic group, these disability diagnoses were relatively uncommon among the highly educated individuals and relatively common in firms with many low educated employees. The total variance explained was high, 38.8%, for men and 25.9% for women. The intraclass correlations suggested that 3.1% and 4.7% of the unexplained variation was attributable to differences between firms for men and women, respectively.

*Complex psychiatric diagnoses* (depression, anxiety and similar conditions, n= 11,558) were common diagnoses for those from firms downsizing 30-59%, especially for women. High education was relatively common among this group of pensioners who were quite evenly distributed across educational levels in firms. For this diagnostic group, our model explained a small part of the total variance, 14.6% for men and 11,3% for women, with intraclass correlations of 0.083 and 0.032 respectively.

*Well-defined psychiatric diagnoses* (psychoses, oligophrenia and others, n = 3,003) seemed to be weakly or not associated with firm downsizing. This diagnostic group was quite evenly distributed according to other firm characteristics and not uncommon among highly educated DPs. Men had more often got DP for specific psychiatric diagnoses than women, and as much as 18.9% of their total variance was explained by the model compared to just 5.2% for women. Of the unexplained variance, 5.4% was attributable to differences between firms for women and 6.8% for men.

*Other somatic diagnoses* was the biggest diagnostic group (n = 27,632) and showed a similar pattern to all diagnoses in Table 3. This group contributed to a middle high disability rate among employees from firms with 30-59% downsizing. The intraclass correlation coefficient was low for women (0.016) but of a reasonable size for men (0.088). The total explained variance was very low for men (2.6%) and higher for women (17.2%), showing that our model did not explain much of these male DP cases with diseases being patterned more by fate than by social conditions. Still, some of them seemed to be influenced by firm characteristics like downsizing. For females, firm characteristics had a greater influence on disability rates with well-defined somatic diseases.

## Discussion

We find that firms downsizing 30 to 59% of their workforces between 1995 and 2000 in Norway have about 25% higher DP rates over the next four years than employees in non-downsizing firms. This is a socially and clinically significant result. Firms downsizing by 60-100% did not contribute to a significant increase in DP rates in this study. This is surprising, partly because of our experiences as GPs giving us the opposite impression, partly because Rege et al. found a different result in an older version of the same dataset [5]. We also find that those who stay in downsizing firms more often get DP than leavers, in contrast to our hypothesis. Diagnosis-specific DP shows an interesting result - what we have called complex diseases more often are used for DPs after downsizing than well-defined diseases.

### Methods

The strengths of the present study are that the dataset comprises all Norwegians in the period 1995 to 2004 with all DPs granted. The demographic variables are very reliable with one important exception: employment. The weakness here is that employment was missing for 25.6% of the population in 1995. Formally, it is obligatory to register all employed persons [10], and this is routinely done by larger firms and official employers but smaller firms will often not do that until an employee is sick-listed for more than 16 days, when it becomes necessary to register that person. Thus, missing cases are not randomly distributed. We find that they often have short education and low income but not so different from those with registered employment. Hence, the analysed sample may not be far from representative for Norwegian employees.

### Results

A marked increase in DP rates after 30-59% downsizing but not after 60-100% may be explained by moderate reduction of the workforce hitting elderly and chronically ill employees more selectively than major reductions. There is a tradition in Norway, however, than those “last in” are the first to go when downsizing, and this is often but not always followed up by the unions. Firms often get around this tradition by offering economic compensation or simply help with applications for a DP to those who leave the job voluntarily.

Rege et al. also found a 24% increase in DP rates in 2001 following 60-100% downsizing in 1995–2000, and significantly a 9% increase after 30-60% downsizing [5]. We used DP rates for a further three years, and the process of getting a DP after minor downsizing may take more than one year. If a redundant worker has a chronic disease or being redundant leads to a disease, for instance depression or low back pain [11], this may lead to a year on sick-pay. If the condition lasts and makes return to work really difficult, the former employee will stay on a rehabilitation grant for one to three years before the NAV officers will decide to give a DP.

The same processes may explain our finding that stayers more often get a DP than leavers. Leavers may often be the “last in” and more healthy than the stayers who more often need time and help from the firm to be able to leave their job.

### Diagnostic groups

Our method of dividing DP diagnoses into “complex” and “well-defined” diseases is new [7,8] but shows its applicability in the present study. We find that those getting a DP after downsizing more often have diagnoses of complex diseases. This does not mean that they are not diseased but that they have problems which reduce their chance of a new job after redundancy. Such ailments are common in working life, and are often compatible with the present job but not with many new jobs at the labour market.

This classification of diagnoses may have low reliability. However, we found it not difficult by following present traditions in Norwegian general practice and common medical thinking. Alcohol and drug diagnoses as the medical reason for disability are legally difficult, adding to our classification as complex diagnoses. Osteoarthritis may be regarded as a “diffuse diagnosis” by the NAV officers and by GPs writing certificates for a DP but is well defined medically and is classified as such here. Some somatic diagnoses are often considered as “diffuse” and “functional” without precise criteria, like gastritis and spastic colon, but these diagnoses were singled out by the GPs not using them on DP certificates. We recommend that social security researchers should use this classification.

### Firm variance

In our study, the intraclass correlation coefficients are small; 3.6% of the total unexplained variance in receiving DP for men and 2.1% for women is attributable to differences between firms. Individual characteristics account for most of the variance, including the well known factors age, education and income [9,12]. This reflects the relatively small impact of firm downsizing but also other firm characteristics like mean income and mean educational level are largely attenuated by the corresponding individual variables (Table 3 Model 3). Given that the intraclass correlation coefficients are higher for men than for women, this is probably a result of a very gender-specific labour market in Norway, women may more often be working in firms with high but different demands of good health and good abilities for manual work. Alternatively, in some firms they may give up working more easily than men, for instance in health and social work. Social security practitioners as well as researchers should be more aware of these gender divedes in the labour market.

## Conclusion

Our study shows that moderate downsizing is followed by a small but significant increase in disability pension rates in the next four years. Those staying in the job afterwards have a higher risk than those leaving. Downsizing especially leads to increased rates of DPs with complex medical diagnoses.

## Pre-publication history

The pre-publication history for this paper can be accessed here:

http://www.biomedcentral.com/1471-2458/13/27/prepub
